# Assessment of Successful qRT-PCR of SARS-CoV-2 Assay in Pool Screening Using Isopropyl Alcohol Purification Step in RNA Extraction

**DOI:** 10.1155/2021/6653950

**Published:** 2021-06-08

**Authors:** Mayank Gangwar, Alka Shukla, Virendra Kumar Patel, Pradyot Prakash, Gopal Nath

**Affiliations:** ^1^Viral Research and Diagnostic Laboratory, Department of Microbiology, Faculty of Medicine, Institute of Medical Sciences, Banaras Hindu University, Varanasi, India; ^2^Department of Gastroenterology, Faculty of Medicine, Institute of Medical Sciences, Banaras Hindu University, Varanasi, India

## Abstract

The study is aimed at establishing the optimal parameters for RNA purification of pooled specimens, in SARS-CoV-2 assay. This research work evaluates the difference of extracted RNA purity of pooled samples with and without treatment with isopropyl alcohol and its effect on real-time RT-PCR. As per the protocol of the Indian Council of Medical Research (ICMR), 5 sample pools were analysed using qRT-PCR. A total of 100 pooled samples were selected for the study by mixing 50 *μ*L of one COVID-19 positive nasopharyngeal/oropharyngeal (NP/OP) specimen and 50 *μ*L each of 4 known negative specimens. Pool RNA was extracted using the column-based method, and 1 set of pooled extracted RNA was tested as such, while RNA of the second set was treated additionally with chilled isopropyl alcohol (modified protocol). Further, the purity of extracted RNA in both the groups was checked using Microvolume Spectrophotometers (Nanodrop) followed by RT-PCR targeting E-gene and RNaseP target. The results showed that the purity index of extracted RNA of untreated pooled specimens was inferior to isopropyl alcohol-treated templates, which was observed to be 85% sensitivity and 100% specificity. The average Cq (E gene) in the unpurified and purified pool RNA group was 34.66 and 31.48, respectively. The nanodrop data suggested that purified RNA concentration was significantly increased with an average value of 24.73 ± 1.49 ng/uL, which might be the reason for high sensitivity and specificity. Thus, this group testing of SARS-CoV-2 cases using pools of 5 individual samples would be the best alternative for saving molecular reagents, personnel time, and can increase the overall testing capacity. However, purity of RNA is one of the important determinants to procure unfailing results, thus, this additional purification step must be included in the protocol after RNA has been extracted using commercially available kit before performing qRT-PCR.

## 1. Introduction

It is a very well-known fact that the global pandemic named COVID-19 caused by a novel severe acute respiratory syndrome coronavirus 2 (SARS-CoV-2) hit the world in late December of 2019. It has enveloped major parts of the world resulting in a global emergency in most countries with more than 163,869,893 confirmed cases and 3,398,302 deaths as per the latest May 2021 COVID19 situation report of WHO [1]. The foremost priority in front of all the healthcare authorities is to facilitate reliable and fast diagnosis of active SARS-CoV-2 infection. Nowadays, many diagnostic methods for COVID19 are available such as imaging (chest CT), Ag/Ab (ELISA based or RAPID kits), and RNA detection (next-generation sequencing, qRT-PCR (real-time reverse transcription-polymerase chain reaction), and LAMP assay) [2]. However, qRT-PCR is still the gold standard among the entire aforementioned diagnostic tools. In the current scenario, increasing sample load for testing RT-PCR created limited nucleic acid-extraction kits and molecular reagents. To meet this exceeding demand of testing capacity, the concept of pool screening has gained spotlight throughout the world. This test requires 5-8 samples, which are mixed and screened in a single pool, and if the pool reflects positivity (i.e., amplified graph), then subsequent individual testing of each sample of that respective pool is recommended. This approach proved to be reliable, faster, and economical [3]. A recent report by Gupta et al. showed that pool screening would significantly decrease the testing time and expenses. Besides, this method would also increase the diagnostic potential by 5 to 8 times [4, 5]. The concept of pool testing was employed in the detection of influenza viruses [6] and in other infectious diseases [7, 8]. However, the success of testing relies on its limit-of-detection, sensitivity, and specificity for samples that were pooled and the disease prevalence in any specific population. Also, the efficacy of RTPCR detection is proportional to the constitution of the RNA extract, which in turn rests on the employed nucleic acid extraction protocol. Since SARS-CoV-2 is a novel pathogen with limited information about its RNA sensitivity, it needs special consideration. In the current pandemic of COVID-19, the reverse transcriptase-polymerase chain reaction (RT-PCR) based qualitative detection of SARS CoV-2, in pooled respiratory specimens, is necessary to deal with many samples for the surveillance of the disease. Further, this is also essential for the early detection of asymptomatic cases to interrupt the chain of transmission of the virus. Initially, we failed to detect the virus in extracted RNA from the pooled specimens of 5 samples, in which only one sample was known positive, using column-based RNA extraction kits employing the steps as described in the kit insert. This may be due to the dilution of RNA template in pooled samples; we tried to detect the virus from the extracted RNA obtained from the pooled samples after treating it with chilled isopropyl alcohol, which yielded the desired results. A novel RNA purification protocol of COVID-19 samples was designed and standardized. Therefore, we decided to evaluate the difference of purity of extracted RNA with and without treatment with isopropyl alcohol and its effect on PCR.

Thus, the present experiment was designed to test the sensitivity of standard qRT-PCR and reevaluated the sensitivity of pool results by addition of RNA purification step using isopropyl alcohol, which could improve the qRT-PCR performance for SARS CoV-2 virus screening.

## 2. Materials and Methods

### 2.1. Sample Selection

**Figure 1:**
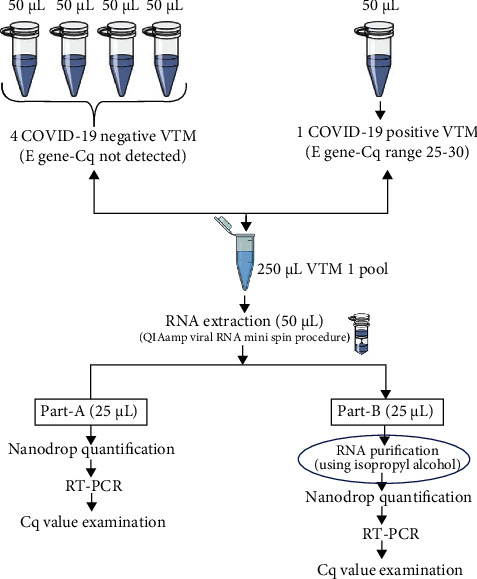
Detailed methodology for selection of COVID-19 pool samples for RNA purification using isopropyl alcohol.

Routine COVID-19 samples received in Viral Research and Diagnostic Laboratory (VRDL) from various health care centres of Varanasi, UP, India, were selected from July to August 2020 (all symptomatic and contact definition populations). Each sample was a combination of nasopharyngeal and oropharyngeal swabs in a 3 mL viral transport media (VTM) preserved under cold chain. A total of 500 samples were selected for the study that includes 100 positive and 400 negatives. The positive COVID-19 sample selected for the study includes Cq values ranging from 25-30, while negative samples were selected without amplification. These samples were pooled in to form 100 pools (each pool containing 5 samples) for RNA extraction. All the samples were received in VRDL, Department of Microbiology, Institute of Medical Sciences, Banaras Hindu University, Varanasi, India. 50 microlitre (*μ*L) VTM of each of the 5 samples was pooled in to form a single pool (Figure 1), and these pools were further processed for viral nucleic acid extraction as per the manufacturer's protocol [8].

### 2.2. Column Based RNA Extraction and Modified RNA Purification Protocol in Pool Testing

All the samples for viral nucleic acid extraction were treated as per the manufacturer's instruction by column-based viral RNA/DNA Mini Kit (QIAamp Viral RNA Mini Kit (Qiagen, Hilden, Germany, Cat. No 52906, Lot no. 166028933)). Extraction control (EC) was also used at the time of RNA extraction in each batch, to check the validity of the extraction procedure. RNAs of 100 pooled samples extracted were divided into two parts; one part with 25 *μ*L RNA was defined as unpurified RNA and was kept as such for qRT-PCR for SARS CoV-2 virus screening assay without purification. However, the remaining template (25 *μ*L RNA) was purified using the isopropyl alcohol method (modified RNA purification protocol) (Figure 1). This RNA purification step included the addition of isopropyl alcohol (50 *μ*L) in eluted RNA, which was placed at -20°C for 15 minutes. Further, the tubes were centrifuged at 14000 RPM for 10 minutes (at 4°C). The supernatant was discarded, and 100 *μ*L of absolute ethanol (Sigma-Aldrich, MB grade) was added and placed at room temperature for 5 minutes. Further, the supernatant was discarded very gently, and the tubes were allowed to dry in a horizontal position for 10 minutes. After drying, 30 *μ*L of AVE (elution buffer) was added and mixed, while 50 *μ*L of elution buffer was used in extraction using the column-based kit.

### 2.3. RNA Measurement/Quantification Using NanoDrop

Isolated purified and unpurified pool sample RNA concentrations/purity were measured using NanoDrop One^C^ equipment (NanoDrop Technologies Inc., Wilmington, DE by Thermo Scientific). The sample absorbance was measured at 260 nm and 280 nm, and the 260/280 ratio was used to assess RNA purity along with the nucleic acid concentrations in ng/mL. As per recommended guidelines, RNA purity was considered adequate when the ratio of 260/280 was ~2.0 [9]. However, deviated ratio values may indicate the presence of proteins, phenol, or other contaminants, which generally show strong absorbance at 280 nm and unusual RNA concentrations [10].

### 2.4. Molecular Detection of COVID-19 Using qRT-PCR Assay

5 *μ*L of extracted RNA (purified and nonpurified) was added to 20 *μ*L of reaction master mix prepared using real-time fluorescent qRT-PCR kit (Invitrogen). SARS-CoV-2 qRT-PCR assay was performed using first-line screening E gene assays (envelope small viral membrane protein) and human ribonuclease P (RNase P, as internal control) gene target, which was included to confirm the authenticity of the specimen collection, RNA extraction, identification of PCR inhibitors, and also defined as internal positive control [11]. Second-line confirmatory assay was performed using RdRp and HKU ORF gene [12]. The primer and probe sequences used for E gene, RdRp, ORF1b, and RNaseP identification are shown in Table 1. ICMR-NIV, Pune provided all the above oligonucleotides along with Standard Operating Procedure for detection of 2019 novel coronavirus (2019-nCoV) in suspected human cases by rRT-PCR.

The values of amplification (Cq) and fluorescence (RFU) were noted and compared for both sets (purified and nonpurified extracted RNAs).

### 2.5. qRT-PCR Amplification Cycle

Superscript III one-step qRT-PCR system with Platinum Taq Polymerase (Invitrogen SuperScript™ III Platinum® One-Step Quantitative Kit from Invitrogen, Darmstadt, Germany) was used for the detection of COVID-19 targets. The reaction mixture composition used for SARS-CoV-2 detection was 12.5 *μ*L of 2 × reaction buffer containing 0.4 mM of each deoxyribonucleotide triphosphate (dNTP) and 3.2 mM magnesium sulphate, 0.5 *μ*L of reverse transcriptase/Taq enzyme mixture provided with kit, 1 *μ*L of E/RNase P gene primer-probe mix, and 6 *μ*L DNase/RNase-free water. Thermal cycling conditions used for screening as well as for confirmatory assay were 55°C for 30 minutes for reverse transcription, followed by 95°C for 3 minutes and then 45 cycles of 95°C for 15 seconds, 58°C for 30 seconds [13]. The assay was performed using CFX96™ Optical Reaction Module using CFX Manager™ Software (Bio-Rad).

### 2.6. Statistical Analysis

The raw values obtained from NanoDrop (A260/A280 ratio) were statistically analysed using chi-square and Mann–Whitney tests in SPSS software. The sensitivity and specificity of the tested samples were evaluated and presented in a 2 × 2 table.

## 3. Results and Discussion

### 3.1. RNA Quantification

A total of 100 pooled specimens of known SARS-CoV-2 positive, along with available negative samples, were selected to assess this proposed RNA purification method. Both sets of extracted RNAs were subjected to spectrophotometric analysis. The average concentration of purified set of pooled RNAs showed a value between 15.5 and 56.8 ng/*μ*L, while the unpurified set of RNAs presented with a range between 1.24 and 8.14 ng/*μ*L. Also, the values of absorbance ratio (at 260/280 nm) of unpurified RNAs were reported between 0.9 and 4.8. In addition, the RNA concentration detected using NanoDrop in purified pooled RNA samples were 24.73 ± 5.60 ng/*μ*L, while in unpurified pooled RNA it was detected to be 2.38 ± 1.09 ng/*μ*L. The A260/A280 ratio for purified RNA was ∼2.0, whereas unpurified RNA presented a higher 260/280 ratio. The results showed that the A260/A280 ratio in unpurified and purified RNA samples was 1.58 ± 0.06 and 2.02 ± 0.02, respectively.

### 3.2. qRT-PCR Analysis

**Figure 2:**
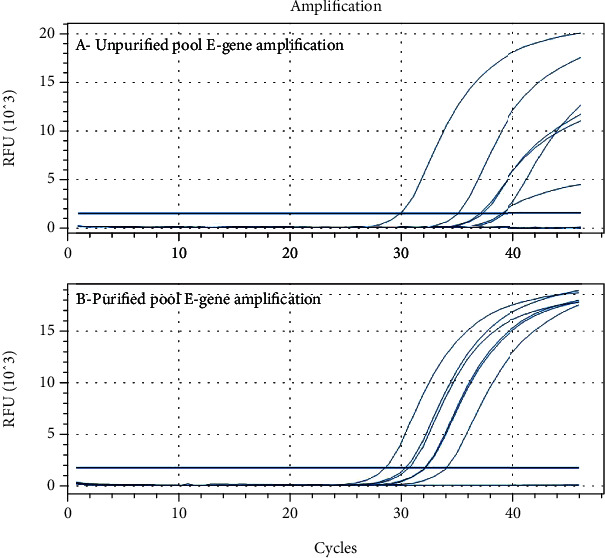
Relative fluorescence units (RFU) and E gene amplification of (a) unpurified RNA of pooled specimen and (b) purified RNA using qRT-PCR 45 cycles.

Next, by employing qRT-PCR, we detected the SARS-CoV-2 E gene and endogenous positive control gene RNase P in both purified and unpurified RNA. Both the sets of RNAs were executed simultaneously, and the qRT-PCR results of E-gene showed successful amplification in both groups. However, one pool did not show significant amplification of E gene in unpurified set (i.e., out of recommended range of detection 35 Cq), while it was significantly amplified in purified set (i.e., detected in recommended borderline range within 35 *C*_*q*_). Similarly, the RNase P gene was also not detected in 15% of the samples. The average *C*_*q*_ values of RNase P in the unpurified pool group was 29.46, while average values after purification were 27.44. However, 15% of the samples (15 pool samples RNA) were reported with very high Cq of RNase P ranges from 37.06 to 41.56, which was considered as inappropriate sample or repeat sampling. But, after purification, the *C*_*q*_ values of RNaseP were 34.05 to 35.37, i.e., in the recommended detectable range. Amplification was successful with lower *C*_*q*_ values after purification. Using modified RNA purification protocol with isopropanol, 75% of the total tested pools showed lower *C*_*q*_ values when compared with the unpurified RNA set. The difference of 2.3 to 4.5 low *C*_*q*_ was observed in purified RNA (Figure 2(b)) when compared with the unpurified set (Figure 2(a)). In addition, the level of fluorescence (RFU) detected in purified was significantly high as compared with the unpurified one (Figure 2). Thus, it can be concluded that successful amplification of SARS-CoV-2 target genes in qRTPCR was detected after incorporating an additional purification step in extracted RNA with isopropanol.

The results also showed that the samples with a high concentration of RNA (50 ng/*μ*L) and high purity index of 1.9 depicted remarkable amplification graphs in both the cases (in unpurified and purified sets of RNA). However, the samples that may contain low viral load and low quantity of RNA showed progressed amplification graphs in the purified set and can be successfully detected after purification. The overall sensitivity and specificity of pool samples before and after purification of extracted RNA was calculated and represented in a 2 × 2 table. The results showed that sensitivity with 85% and specificity found to be 100% among 500 samples. In addition, PPV (positive predictive value) and NPV (negative predictive value) were found to be 100% and 96.38%, respectively. The details of true or false positive or negative were tabulated in Table 2. This could be attributed to the fact that the purification process removes most of the inhibitory factors/impurities associated with the RNA and organic substances. These impurities are responsible for high *C*_*q*_ values (near borderline ~36), making the lab person declare the respective pool as negative, which might have one or two suspected cases.

The statistical analysis demonstrated that the RNA purification step with the isopropanol method significantly increased the amplification success rate of SARS-CoV-2 detection in pool testing. That could be due to the less sample volume in pool testing (50 *μ*L) as compared with individual RNA isolation (~250 *μ*L). Besides, the purification step resulted in an improved quantity and quality of RNA for COVID-19 pool screening.

## 4. Discussions

The study showed that the purification of RNA using a modified isopropanol step could be a critical step to report true positives. Pooling of samples and purification of RNA can enhance the diagnostic efficiency during pandemic screening [14–16]. Thus, this study was undertaken to ensure the purity of RNA, which will decide the depooling or individual testing of included samples for the SARS CoV-2 virus. Key factors, which determine the successful application of pool testing, involve limit-of-detection, RNA purity, sensitivity, and specificity. However, as per WHO criteria, any fluorescence after 40 cycles should not be considered positive [17]. The purification process used in this study increased the chances of getting amplification with Cq values under the recommended range. The impurities/amplification inhibitors present in the extracted RNA of pooled specimens might be one of the reasons for false negativity along with other factors such as inappropriate collection, transportation, or improper handling before processing. However, varying viral-load kinetics of SARS-CoV can be affected by sampling timing and period of disease development. In the present study, an attempt was made to minimize the impurities in obtained RNA, which is the laboratory-based factor. However, we suggest that this modified RNA purification protocol be validated by few other labs in the country to give more strength to the above reported COVID-19 pool test results.

However, it was reported that qRT-PCR based diagnostics have been challenged with naturally occurring inhibitory substances (as reported in diagnosis of avian influenza), which can only be copurified using modified RNA protocol reported by Das et al., 2006 [18]. However, currently available commercial kits may fail to completely remove the qRT-PCR inhibitors in the sample [19], which can be one of the reasons of high rate of false negativity. It was also reported that washing again with wash buffer and enhancing the volume of lysis buffer may increase the chance of amplification in qRT-PCR. The chemistry used in RNA isolation was column-based silica extraction, and the modified purification protocol after extraction showed a significant chance of amplification as compared with the unpurified RNA from the commercial kit. These impurities could be reducing agents, high salts, low pH, etc. [20], which might be forming complexes, that in turn delay the RNA detection in qRT-PCR within the detectable range. However, this additional step of 30-45 mins reduces the chance of reporting false negativity of COVID-19 samples.

In addition to the concentration of extracted RNA obtained from NanoDrop, sample quality can also be gauged by analysing the A260/280 ratio. It was reported that the pure nucleic acids mostly yield a 260/280 ratio of approximately 2 for the RNA sample. However, A260/280 ratio is dependent on the pH and ionic strength of the buffer used in the sample measurements. The ratio below the standards represents the acidic or basic solutions, which indicate protein, phenol, or other contaminants present after extraction that absorbs strongly at or near 280 nm. The purity ratio may vary from the expected values, which indicate the different RNA isolation procedure, which further required optimization before performing a qRT-PCR diagnostic assay to minimize the false-negative results.

## 5. Conclusions

An alternative approach of RNA purification for pooled specimens was designed to obtain error-free results. Application of isopropanol-based purification after extraction of pooled-specimens RNA from commercial kits fetched a high-quality RNA from NP/OP COVID-19 suspected samples, and it could easily be performed in the laboratory. Extracted RNA of the pooled specimen with minimal impurities would be an ideal template for qRT-PCR assay. Thus, proper sampling of COVID-19 suspects, good laboratory practice (GLP) standard, and high-quality extraction procedures with purification using isopropanol would improve real-time RT-PCR findings and reduce inaccurate results.

## Figures and Tables

**Table 1:** SARS-CoV-2 novel coronavirus primers and probes for real-time RT-PCR diagnosis.

Assay/use	Oligonucleotide ID	Sequence (5′–3′)
E gene	E_Sarbeco_F1	ACAGGTACGTTAATAGTTAATAGCGT
	E_Sarbeco_R2	ATATTGCAGCAGTACGCACACA
	E_Sarbeco_P1	FAM-ACACTAGCCATCCTTACTGCGCTTCG-BHQ

RNase P gene (internal control)	RNase P forward	AGATTTGGACCTGCGAGCG
	RNase P reverse	GAGCGGCTGTCTCCACAAGT
	RNase P probe	FAMTTCTGACCTGAAGGCTCTGCGCGBHQ

RdRp gene	RdRP_SARSr-F2	GTGARATGGTCATGTGTGGCGG
	RdRP_SARSr-R1	CARATGTTAAASACACTATTAGCATA
	RdRP_SARSr-P2 specific for Wuhan-CoV	FAM-CAGGTGGAACCTCATCAGGAGATGC-QSY

HKU ORF gene	HKU-ORF1b-nsp14F	TGGGGYTTTACRGGTAACCT'
	HKU-ORF1b-nsp14 R	AACRCGCTTAACAAAGCACTC
	HKU-ORF1b-nsp14 P	FAM-TAGTTGTGATGCWATCATGACTAG-QSY

R is G/A; FAM: 6-carboxyfluorescein; BHQ: Black Hole Quencher; QSY Quencher (select quencher none in plate set up).

**Table 2:** Sensitivity and specificity performance of RNA purification in SARS-CoV-2 assay.

RT-PCR	RNA purification			Sensitivity = 85%
	Positive	Negative	Total	
Positive	85 (TP)	15 (FN)	100	Specificity = 100%
Negative	0 (FP)	400 (TN)	400	PPV = 100%
Total	85	415	500	NPV = 96.38%

PPV: positive predictive values; NPV: negative predictive values; (PPV) positive predictive value: TP/(TP + FP) × 100; (NNV) negative predictive value: TN/(TN + FN) × 100; TP: true positive; FN: false negative; FP: false positive; TN: true negative.

## Data Availability

The datasets generated, analysed, and presented are available in the manuscript, while raw dataset used to support the findings of this study are available from the corresponding author upon request on reasonable request.
